# Ruptured Appendicitis Leading to Development of a Tubo-Ovarian Abscess in a Non-sexually Active Adolescent Patient

**DOI:** 10.7759/cureus.41226

**Published:** 2023-06-30

**Authors:** Angel Hsu, Brianna K Sarowa, Shahab F Abdessalam

**Affiliations:** 1 General Surgery, Rutgers University New Jersey Medical School, Newark, USA; 2 Pediatric Surgery, Newark Beth Israel Medical Center, Newark, USA

**Keywords:** pediatric acute appendicits, mimicker of acute appendicitis, tubo-ovarian abscess nonsexually active, ruptured appendicitis, pediatric tubo-ovarian abscess

## Abstract

Tubo-ovarian abscesses (TOA) are commonly associated with pelvic inflammatory disease (PID) caused by sexually transmitted infections (STI). There have been several reports of adolescent non-sexually active female patients diagnosed with TOAs. Symptoms of TOAs often mimic appendicitis and have often been diagnosed as such. We present a case of a 12-year-old non-sexually active adolescent who was initially diagnosed with ruptured appendicitis and found to have a TOA engulfing the appendix.

## Introduction

A tubo-ovarian abscess (TOA) is an infection of the fallopian tubes and ovaries which often results in abscess formation [1]. Most TOAs occur in the setting of sexually transmitted pelvic inflammatory disease (PID), most commonly due to N. gonorrhea or C. trachomatis [2]. Approximately 15-35% of women diagnosed with PID will have TOAs [3]. But TOAs due to bacterial spread from appendicitis or other bowel disease result in polymicrobial infections [1]. There have been several cases of TOAs in non-sexually active females and around two-thirds of the patients were in the pediatric group [2]. However, the incidence of pediatric TOA is still rare. In a large retrospective study focusing on patients from 2002 to 2014 (n=122), only five non-sexually active women under the age of 24 were found to have TOAs [4]. Patients with TOA often have abdominal pain, fevers, nausea, vomiting, diarrhea, and other symptoms that are also seen in appendicitis. There is often difficulty in differentiating between right-sided TOAs and ruptured appendicitis with abscess on imaging and pediatric patients are often diagnosed with perforated appendicitis instead. 

## Case presentation

We present a case of a 12-year-old female who was transferred from an outside hospital for two days of abdominal pain. She had associated nausea, vomiting, and diarrhea. She was reported to have a white blood cell count of 36 with an 88% left shift and was tachycardic and febrile up to 100.3F but with normal blood pressure. CT scan showed a 9cm multiloculated pelvic mass versus collection. The pediatric surgery team had been initially consulted for perforated appendicitis. Upon further questioning, the patient had started menstruating two years ago and her last menstrual period was about a month ago. She denies being sexually active. The patient admitted that she has had similar episodes of pain over the last three months, although not as severe as this. On exam, the patient had minimal suprapubic tenderness and her abdomen was soft and non-distended with no rebound or guarding. 

**Figure 1:**
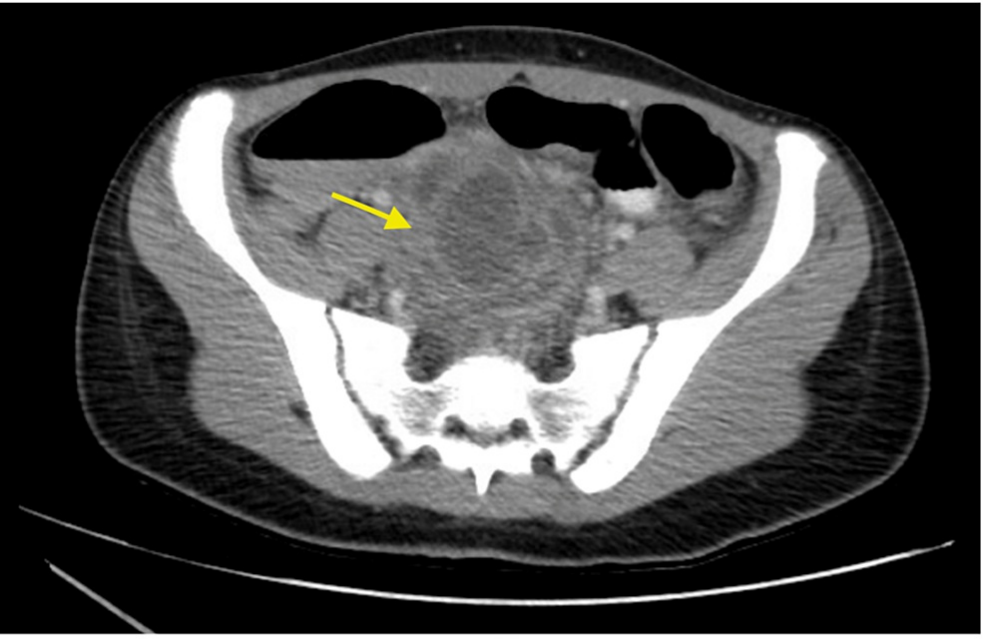
CT Axial view of the right lower quadrant pelvic mass

**Figure 2:**
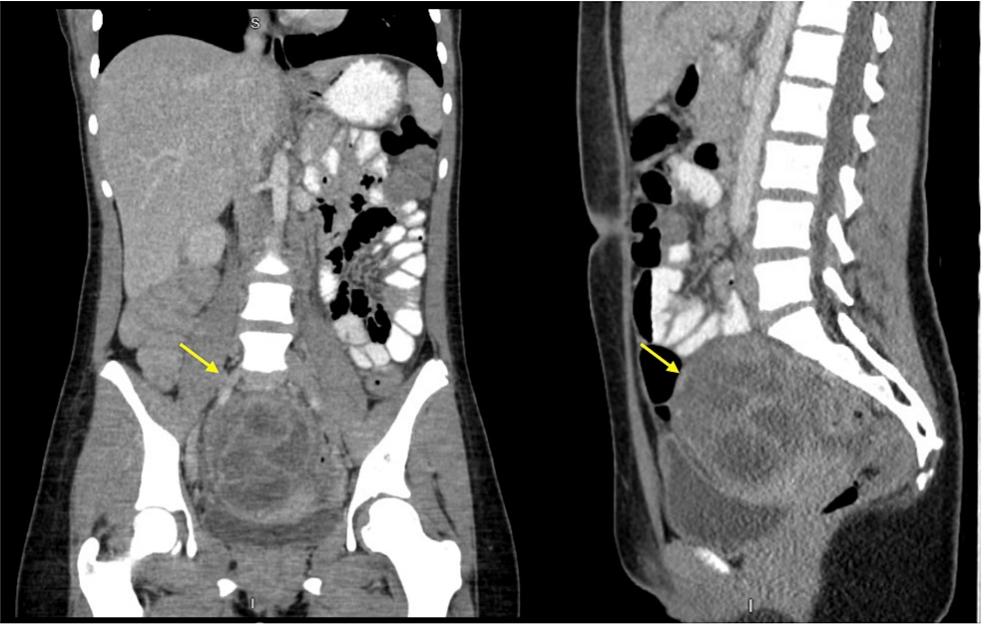
Coronal and sagittal views of the loculated pelvic lesion compressing the bladder

The CT of the abdomen and pelvis showed a multiloculated 8.3 x 6.9 x 8.8cm mass in the central pelvis with enhancing rim, most likely a multiloculated abscess (Figure 1, 2). The uterus is deviated to the left, inferior to the mass, and ovaries are not visualized. Diagnostic considerations include complicated appendicitis with rupture and abscess, tubo-ovarian abscess, or cystic ovarian neoplasm.

The decision was made to proceed with a diagnostic laparoscopy given the diagnostic uncertainty. The appendix could not be identified which raised suspicion of ruptured appendicitis with abscess, but it could also be an ovarian or tubal cystic mass that had subsequently become infected. The mass also had too many septations for percutaneous drainage.

**Figure 3:**
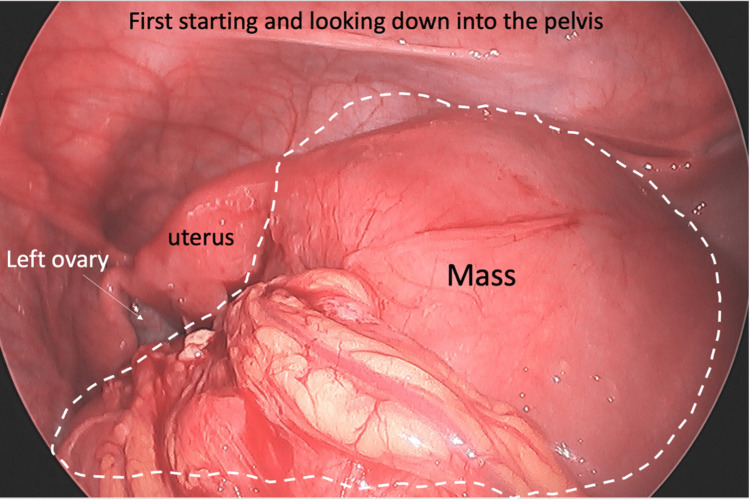
Initial view of the pelvic mass upon entry into the pelvis

**Figure 4:**
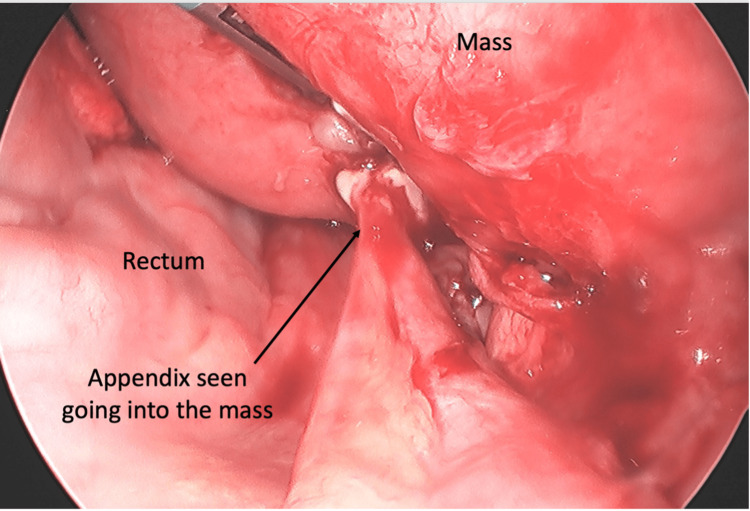
The appendix seen going into the mass

**Figure 5:**
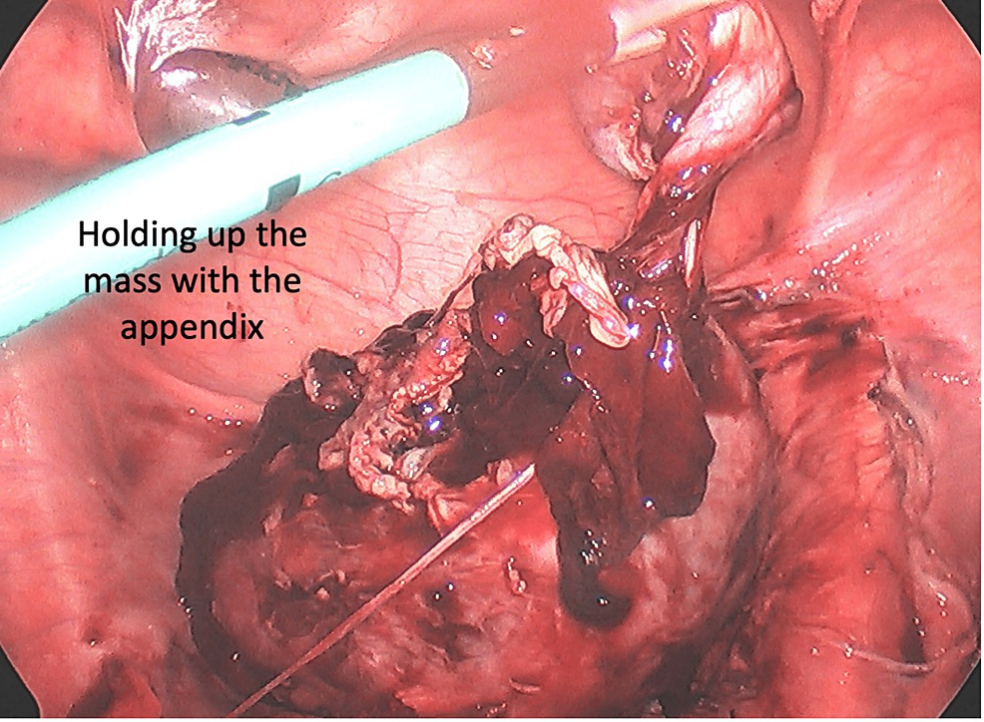
The appendix tip is seen engulfed within the tubo-ovarian mass

**Figure 6:**
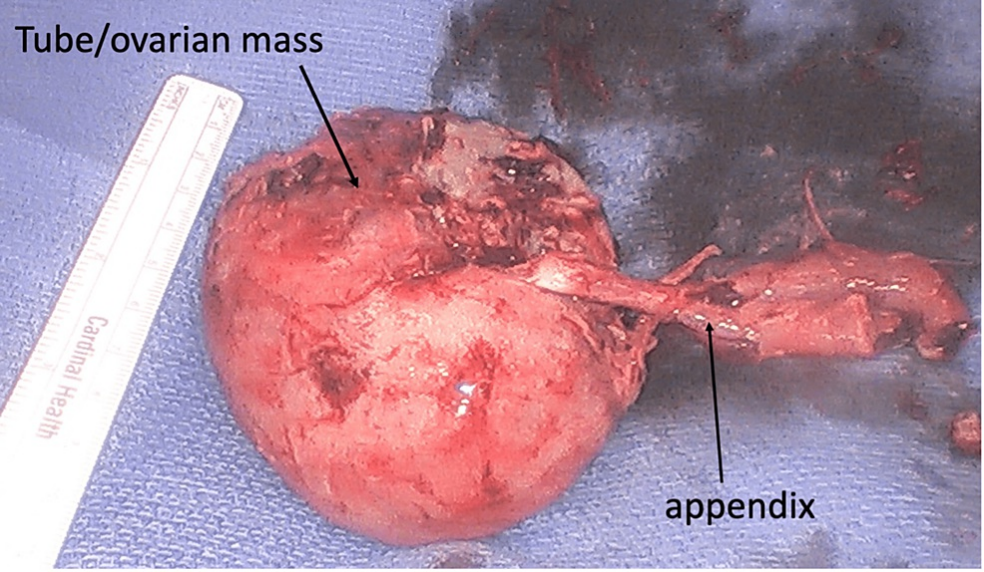
The appendix is attached to the tubo-ovarian mass

In the operating room, the patient was found to have a large right multicystic pus-filled ovarian mass with the appendiceal tip engulfed within the mass (Figure 3-5). The mass was filled with multiple loculations of foul-smelling purulent material. No definitive right ovary could be identified and only the origin of the right fallopian tube could be identified. The pathology report showed a tubo-ovarian abscess with acute suppurative inflammation and xanthogranulomatous inflammation with the distal end of appendix adherent to the tubo-ovarian mass via dense fibrous adhesions (Figure 6). Intraoperative cultures speciated as Eikinella corrodens and Odoribacter splachnicus. The patient did well postoperatively and was discharged home one day after. 

## Discussion

There have been several cases of pediatric TOAs mimicking appendicitis. Cheong reports a 13-year-old non-sexually active post-menarchal female who also presented with fever, anorexia, vomiting, and fluctuating abdominal pain [2]. Ultrasound revealed a large retro-uterine heterogeneous collection measuring 10 x 8 x 11.4cm mass that was thought to be a ruptured appendix with abscess [2]. The patient subsequently underwent laparoscopy, and the mass was identified as a left tubo-ovarian abscess [2]. Her appendix, however, was uninvolved [2]. Fei et al. looked at multiple case reports of TOAs in non-sexually active females (n=43) and found that most of the patients presented with abdominal pain, nausea, fever, diarrhea, dysuria, but none of them had vaginal itching, pain, or discharge [1]. Initially 60% of the patients were treated conservatively with empiric antibiotics but half of the patients had worsening symptoms and required surgical intervention [1]. In 50% of the patients, the appendix was involved with the TOA and thought to be the possible source of infection [1]. In another retrospective study, Serbanescu et al. found that three out of 25 patients diagnosed with right and left TOA also had appendicitis and Nishida et al. cited a case of a 12-year-old virginal female who developed recurrent TOAs five years after an appendectomy [5,6]. 

While most TOAs are associated with sexually transmitted infections (STI) and PID, causes of TOAs in non-sexually active females include bowel translocation, urinary infection, or genitourinary anomalies [6]. Imaging can offer helpful insights to differentiate between TOA and other etiologies such as appendicitis, but in several cases, the definitive diagnosis was made in the operating room via exploratory laparotomy [6]. Hakim et al. suggests that appendicitis can result in the development of TOAs by bacterial translocation or direct spread [7]. In a case series of 16 non-sexually active females with TOAs, all patients tested negative for sexually transmitted infections and 76% had either recent appendicitis or genitourinary abnormalities [7]. Intra-abdominal adhesions from ruptured appendicitis can also cause adherence of bowel to the adnexa and fallopian tubes enabling bacterial translocation [6]. In a retrospective study done by Margaux Becker et al., intra-abdominal adhesions from appendicitis is a risk factor for tubal obstruction and infertility [8]. The fallopian tubes also secrete more nutrients and hormones during the menstrual cycle, so combined with adhesions or active appendicitis, can cause development of TOAs [6]. Other factors such as recurrent urinary tract infections, inflammatory bowel disease, or obesity are also known to contribute to the development of TOAs [4]. 

In our case, the tip of the appendix was found to be completely engulfed within the TOA. While it is not entirely clear the etiology of her TOA, the most likely explanation is acute appendicitis which spread to the right ovary and fallopian tube resulting in TOA. The appendix was completely adhered within the TOA and intraoperative cultures speciated as Eikinella corrodens and Odoribacter splachnicus which are associated with the gastrointestinal tract. Eikinella corrodens is a gram-negative rod bacterium that is found in the oral cavity but has been isolated in phlegmonous collections in children who have acute appendicitis [9]. Orodibacter splachnicus is a bacteria found in the intestinal microbiome [10]. 

## Conclusions

Appendicitis seems to be associated with TOAs found in non-sexually active female patients. Patients often require surgical intervention for both definitive diagnosis and treatment. Our patient likely had appendicitis which resulted in the development of a TOA. While TOAs are not common in the pediatric population, it is still important to keep in the differential, especially with the higher incidence of appendicitis seen in this age group. 

## References

[1] Fei YF, Lawrence AE, McCracken KA (2021). Tubo-ovarian abscess in non-sexually active adolescent girls: a case series and literature review. J Pediatr Adolesc Gynecol.

[2] Cheong LH, Emil S (2013). Non-sexually transmitted tubo-ovarian abscess in an adolescent. J Pediatr Surg Case Rep.

[3] Campbell M, Noor AA, Castaneda M (2021). A case of tubo-ovarian abscess in a 15-year-old female after appendectomy complicated by peritonitis. Cureus.

[4] Fink D, Lim PP, Desai A, Stephans AB, Wien MA (2022). Recurrent tubo-ovarian abscess in a nonsexually active adolescent. Consultant.

[5] Serbanescu L, Badiu D, Popescu S (2021). The management of tubo-ovarian abscesses associated with appendicitis. J Mind Med Sci.

[6] Nishida N, Shono T, Shono K, Hashimoto Y, Kawakami K (2022). Late occurrence of the tubo-ovarian abscess after appendectomy for perforated appendicitis in a virginal adolescent girl. J Pediatr Adolesc Gynecol.

[7] Hakim J, Childress KJ, Hernandez AM, Bercaw-Pratt JL (2019). Tubo-ovarian abscesses in nonsexually active adolescent females: a large case series. J Adolesc Health.

[8] Margaux Becker V, Silver S, Seufert R, Muensterer OJ (2019). The Association of appendectomy, adhesions, tubal pathology, and female infertility. JSLS.

[9] Blod C, Schlichting N, Schülin S (2018). The oral microbiome-the relevant reservoir for acute pediatric appendicitis?. Int J Colorectal Dis.

[10] Hiippala K, Barreto G, Burrello C (2020). Novel Odoribacter splanchnicus strain and its outer membrane vesicles exert immunoregulatory effects in vitro. Front Microbiol.

